# Difference in mortality rates in hospitalized COVID-19 patients identified by cytokine profile clustering using a machine learning approach: An outcome prediction alternative

**DOI:** 10.3389/fmed.2022.987182

**Published:** 2022-09-20

**Authors:** Ana Cristina Castro-Castro, Lucia Figueroa-Protti, Jose Arturo Molina-Mora, María Paula Rojas-Salas, Danae Villafuerte-Mena, María José Suarez-Sánchez, Alfredo Sanabría-Castro, Carolina Boza-Calvo, Leonardo Calvo-Flores, Mariela Solano-Vargas, Juan José Madrigal-Sánchez, Mario Sibaja-Campos, Juan Ignacio Silesky-Jiménez, José Miguel Chaverri-Fernández, Andrés Soto-Rodríguez, Ann Echeverri-McCandless, Sebastián Rojas-Chaves, Denis Landaverde-Recinos, Andreas Weigert, Javier Mora

**Affiliations:** ^1^Centro de Investigación en Enfermedades Tropicales (CIET), Universidad de Costa Rica, San José, Costa Rica; ^2^Centro de Investigación en Cirugía y Cáncer (CICICA), Universidad de Costa Rica, San José, Costa Rica; ^3^Centro de Investigación en Hematología y Trastornos Afines (CIHATA), Universidad de Costa Rica, San José, Costa Rica; ^4^Unidad de Investigación, Hospital San Juan de Dios CCSS, San José, Costa Rica; ^5^Departamento de Farmacología, Facultad de Farmacia, Toxicología y Farmacodependencia, Universidad de Costa Rica, San José, Costa Rica; ^6^Servicio de Neumología, Hospital San Juan de Dios CCSS, San José, Costa Rica; ^7^Unidad de Cuidados Intensivos, Hospital San Juan de Dios CCSS, San José, Costa Rica; ^8^Servicio de Oncología Médica, Hospital México CCSS, San José, Costa Rica; ^9^Faculty of Medicine, Institute of Biochemistry I, Goethe-University Frankfurt, Frankfurt, Germany; ^10^Frankfurt Cancer Institute, Goethe-University Frankfurt, Frankfurt, Germany; ^11^Cardio-Pulmonary Institute (CPI), Frankfurt, Germany; ^12^German Cancer Consortium (DKTK), Partner Site Frankfurt, Frankfurt, Germany

**Keywords:** COVID-19, SARS-CoV-2, CXCL10, IL-38, cytokine profile

## Abstract

COVID-19 is a disease caused by the novel Coronavirus SARS-CoV-2 causing an acute respiratory disease that can eventually lead to severe acute respiratory syndrome (SARS). An exacerbated inflammatory response is characteristic of SARS-CoV-2 infection, which leads to a cytokine release syndrome also known as cytokine storm associated with the severity of the disease. Considering the importance of this event in the immunopathology of COVID-19, this study analyses cytokine levels of hospitalized patients to identify cytokine profiles associated with severity and mortality. Using a machine learning approach, 3 clusters of COVID-19 hospitalized patients were created based on their cytokine profile. Significant differences in the mortality rate were found among the clusters, associated to different CXCL10/IL-38 ratio. The balance of a CXCL10 induced inflammation with an appropriate immune regulation mediated by the anti-inflammatory cytokine IL-38 appears to generate the adequate immune context to overrule SARS-CoV-2 infection without creating a harmful inflammatory reaction. This study supports the concept that analyzing a single cytokine is insufficient to determine the outcome of a complex disease such as COVID-19, and different strategies incorporating bioinformatic analyses considering a broader immune profile represent a more robust alternative to predict the outcome of hospitalized patients with SARS-CoV-2 infection.

## Introduction

In December 2019, a novel Coronavirus was described in a cluster of patients with pneumonia of unknown etiology that was epidemiologically linked to Wuhan, Hubei Province in the People’s Republic of China (1). The World Health Organization (WHO) declared this new virus which causes coronavirus Disease 2019 (COVID-19) as a pandemic on March 11th, 2020. COVID-19 is a disease caused by the zoonotic novel coronavirus SARS-CoV-2 which can cause an acute respiratory disease that can eventually lead to severe acute respiratory syndrome (SARS) (2). According to the WHO, at the end of July 2022 there are 571 239 451 confirmed cases of COVID-19 worldwide with a mortality rate of 1.12%.

The first case in Costa Rica was reported on March 6th, 2020.^1^ At the beginning of July 2022, a total of 904 934 confirmed positive cases have been reported with an overall case-fatality rate of 1.1% (Ministerio de Salud, Gobierno de Costa Rica). COVID-19 has become one of the most common causes of death within the last year, especially among the elderly, immunocompromised patients, and patients with multiple comorbidities or multiple chronic conditions, including cardiovascular disease, hypertension, cerebral infarction, and respiratory illnesses (1–3). Importantly, the clinical characteristics and the immune factors associated with mortality have not been addressed in Central America.

The clinical manifestations of COVID-19 appear after an incubation period of 5–6 days and most frequently includes fever, coughing, and fatigue, with the possible onset of sputum production, headache, hemoptysis, diarrhea, dyspnea, and/or lymphopenia, among others (4, 5). Most patients with COVID-19 experience mild self-limited disease (6); however, up to 20% of known cases have suffered from severe pneumonia and consequent acute respiratory distress syndrome (ARDS) after an average of 8–9 days from the onset of initial symptoms (5–7). In severe cases, ARDS causes acute hypoxemic respiratory failure, an alteration that has being identified in 70% of fatal COVID-19 cases (5).

An effective screening tool is essential to identify patients with high risk of developing severe illness with higher mortality rates. Several inflammation-associated markers such as C-reactive protein (CRP), ferritin, fibrinogen, D-dimer, neutrophil levels, urea in blood, and the cytokines IL-6, IL-10, and TNF-α have being associated with COVID-19 progression and play an important role in the development of acute lung injury (4, 6, 8, 9). Therefore, several studies aimed to identify epidemiological and clinical factors that can predict the severity of the disease (10). Most severe COVID-19 cases exhibit an exacerbated inflammatory response, leading to a cytokine release syndrome (CRS) also known as cytokine storm. This severely over-reactive immune system causes hyper-inflammation, septic shock complications, vascular disorders, and organ failure. Since the severity of the disease has been associated with the aforementioned cytokine storm (11, 12), different strategies to screen and modulate cytokine responses have been proposed to decrease SARS-CoV-2 infection mortality rate (3, 7, 13–16).

Even though vaccines have reduced the likelihood of suffering serious infection and hospitalization, it is important to consider the “potential appearance of new variants with the capacity to escape vaccine-induced immune responses and/or the reduction of the protective immunity over time.” Therefore, it is still necessary to develop accurate models for the prediction, detection, and monitoring of severe cases (7). Data originated during the current pandemic supports the use of immunomodulatory therapies to control the inflammatory process such as IL-6 receptor antagonists or systemic corticosteroid therapy in patients with severe COVID-19 (17). This suggests that intervention and modulation of CRS are crucial in order to reduce the morbidity and mortality rates in severe COVID-19 patients (3). However, even though single inflammation markers have been associated with COVID-19 severity, analyses considering several cytokines and immune cell populations have been more useful in the classification and prediction of severity and chronicity of COVID-19 (18, 19).

Taking into consideration the importance of the cytokine storm in the immunopathology of the disease, this study analyzes data from COVID-19 hospitalized patients admitted to San Juan de Dios Hospital, the largest adult tertiary referral hospital of the Social Security Health System in Costa Rica and identifies differences in mortality performing unsupervised clustering of patients using a machine learning approach based on their cytokine profile.

## Materials and methods

### Patients

This is a retrospective study of data collected from 194 adult patients admitted to San Juan de Dios Hospital of the Costa Rican Social Security Fund (Caja Costarricense de Seguro Social, CCSS) with positive COVID-19 diagnosis, between April and August of 2020 with at least one sample analyzed for cytokine concentration during the time of hospitalization. This protocol (R020-SABI-00262) was approved by the corresponding Ethics Committee and carried out according to international guidelines and the Costa Rican regulations. Clinical characteristics of the patients (Frequency of symptoms and co-morbidities) are displayed in Supplementary Table 1.

### Data collection

Clinical information was collected from the patient’s medical records and includes age, diagnosis, patient survival after hospitalization, respiratory support requirement, clinical laboratory parameters (Prothrombin time, Partial Thromboplastin Time, C-reactive protein, D-Dimer, Fibrinogen, Procalcitonin, Ferritin, Blood count).

### Quantification of cytokines

A cytokine panel was measured in the University of Costa Rica (CIET-CIHATA-CICICA) in collaboration with Hospital San Juan de Dios. This cytokine panel was measured by our group during the time of hospitalization for each patient as part of their standard care. Flex set Cytometric Bead Arrays (BD Biosciences) (IL-1 beta, IL-6, IL-8, IL-10, IL-17, TNF, IFN-alpha, IFN-gamma, CXCL10, CCL2, CCL3, G-CSF) was used to determine cytokine concentrations according to manufacturer’s instructions. IL-38 concentration was measured using ELISA as previously described (20).

### Data analysis using machine learning

Metadata (epidemiological, demographic, and clinical information) and quantification values of cytokines and laboratory parameters were considered for the machine learning approach. All the analyses were performed with custom scripts in the RStudio software (Version 1.1.453^2^) with the R software (Version 3.6.3)^3^. The following packages were used: ‘‘caret,’’ ‘‘RColorBrewer,’’ ‘‘ggfortify,’’ ‘‘cluster,’’ ‘‘ggpubr,’’ ‘‘survival,’’ and ‘‘pdftools’’ (details^4^).

For the data analysis, 194 patients were included. Due to the differences in the number of time points of laboratory tests for each patient (range 1–9 days after hospitalization), only the median value of each cytokine or laboratory parameter was considered to compare patients. Thus, a machine learning approach was used to create cytokine profiles using an unsupervised clustering strategy (194 patients × 13 cytokines). Hierarchical Clustering (HC), a clustering algorithm, was implemented to define groups based on the similarity of the cytokine values. The parameters of the model were standardized by the dissimilarity metric (Euclidean, Binary, Maximum, Manhattan, and Minkowski) and the expected number of clusters using the Elbow criterion (21), as detailed in Molina-Mora et al. (22). Thus, Maximum distance and *k* = 3 main clusters were selected as parameters of the final model, with the exclusion of cases with a non-consensus profile of cytokines into a sink group. Afterward, clusters were compared using demographic, clinical, and laboratory data with the subsequent statistical tests.

Statistical tests: For comparison between categorical variables (such as Clusters, clinical classification), Chi-square analysis was used. For comparison of quantitative parameters among the categorical variables, ANOVA and Tukey test were used as appropriate. Log-rank test was implemented to compare the survival distributions among cluster in the Kaplan–Meier survival curve.

## Results

### Participants general information and clustering

**FIGURE 1:**
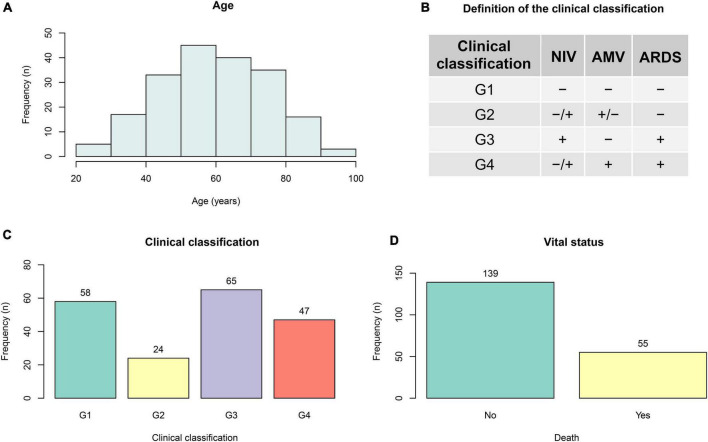
General information. Data from 194 confirmed cases of hospitalized COVID-19 patients from 25 to 97 years **(A)** old were collected, including 55 deaths **(D)** during the time of hospitalization. A clinical classification (G1–G4) was created according to diagnosis of acute respiratory distress syndrome (ARDS), as well as usage of assisted mechanical ventilation (AMV) and/or non-invasive ventilation (NIV) **(B,C)**.

Data obtained from 194 participants were analyzed in the present study, including patients from a broad age range (25–97 years old) showing a normal distribution (Figure 1A). Considering the information available and expert-based criteria by physicians, a clinical classification (G1-G4) was created according to the diagnosis of acute respiratory distress syndrome (ARDS), as well the need for assisted mechanical ventilation (AMV) and/or non-invasive ventilation (NIV) (Figures 1B,C). Of the 194 participants included in this study, 55 died during hospitalization (Figure 1D).

**FIGURE 2:**
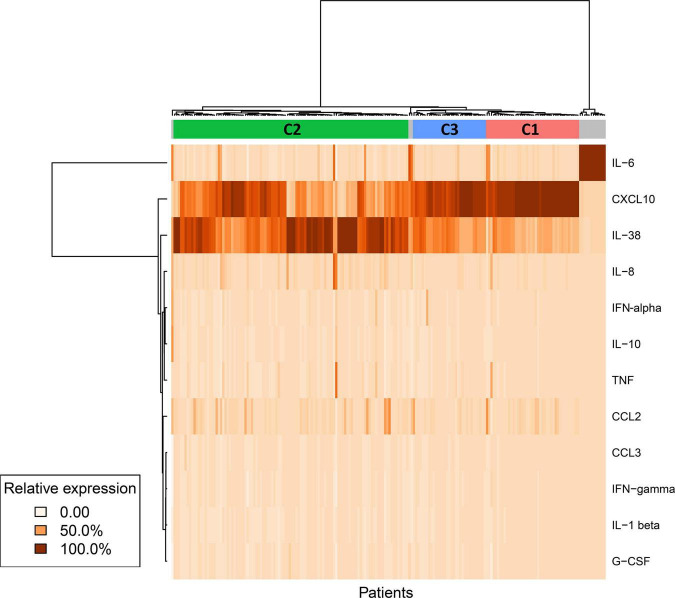
Cytokine profile clustering. Using a machine learning approach, clustering of participants was performed based on their cytokine profile. Median value for each cytokine was used to create profiles or clusters (194 patients × 13 cytokines). The algorithm Hierarchical Clustering (HC) was implemented to define groups based on the similarity of the values of cytokines.

Using a machine learning approach, a clustering of patients was performed based on their cytokine profile (Figure 2). Considering the median value of each cytokine measurement, a total of 3 clusters were identified (C1: 33 patients, C2: 106 patients, C3: 42 patients) in addition to one group of non-fitted participants (sink).

### Cytokine profile and mortality

**FIGURE 3:**
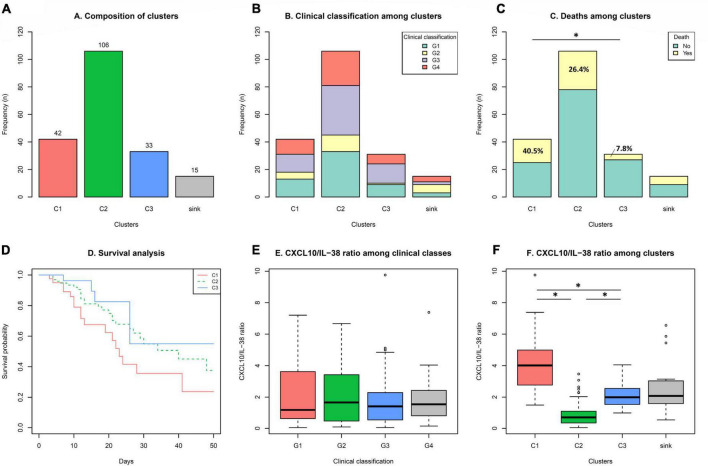
Clinical classification and mortality among the clusters. Considering cluster compositions **(A)**, analysis of: clinical classification **(B)**, mortality **(C)**, and survival **(D)** among the clusters was performed. CXCL10/IL-38 ratio in the clinical classification groups **(E)** and among the clusters **(F)** was analyzed. Statistical analysis using Chi-square was performed excluding the sink group. *Significant differences were found in: Mortality C1 vs. C3 *p* < 0.05; CXCL10/IL-38 ratio among clusters C1 vs. C2 vs. C3 *p* < 0.001.

Analyzing the composition (Figure 3A) and clinical characteristics (Figure 3B) among the clusters (cytokine profiles), no differences were observed regarding the clinical classification defined in this study. However, statistically significant differences in the mortality rate among the clusters (C1 vs. C3) were found (Figure 3C). Specifically, the percentage of mortality was 40.5% in C1, 26.4% in C2 and 7.8% in C3. Additionally, a clear tendency toward a higher survival probability is observed in C3 compared to C1 (Figure 3D). The mortality rate within the clinical classification groups was: G1 5.2%, G2: 29.2%, G3: 15.4%, G4, 74.5%. Interestingly, even though the complete 13-Cytokine profile was used to generate the clusters, considering the two main cytokines in terms of concentration, no differences in the CXCL10/IL-38 ratio was observed between the groups with different clinical characteristics (Figure 3E). Nevertheless, significant differences were obtained in the CXCL10/IL-38 ratio between the clusters, with a higher ratio in the high mortality cluster C1, a lower ratio in the C2, and a middle ratio in the low mortality cluster C3 (Figure 3F).

### Cytokine concentrations and clinical laboratory parameters

**FIGURE 4:**
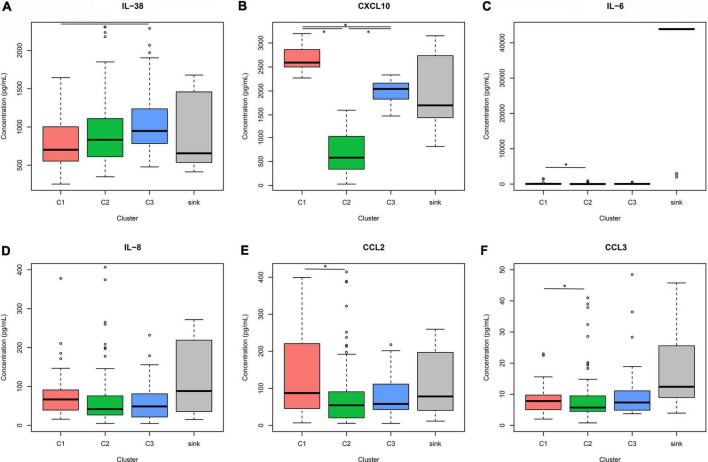
Cytokine concentrations among clusters. Analysis of the concentration values of IL-38 **(A)**, CXCL10 **(B)**, IL-6 **(C)**, IL-8 **(D)**, CCL2 **(E)**, and MIP-1 alpha **(F)** among the clusters was performed. Statistical analysis using ANOVA and Tukey test was performed excluding the sink group. *Significant differences were found in IL-38 concentration C1 vs. C3 *p* < 0.01; CXCL10 concentration C1 vs. C2 vs. C3 *p* < 0.001; IL-6, CCL2, CCL3 concentrations C1 vs. C2 *p* < 0.05.

While evaluating individual cytokine concentrations among clusters a significant difference was observed with the anti-inflammatory cytokine IL-38 between C1 and C3 (Figure 4A), showing higher levels of IL-38 in the low mortality cluster C3. Besides, significant differences were observed in the concentration of the pro-inflammatory chemokine CXCL10 in the three groups, with higher levels in C1 compared with C2 and C3 (Figure 4B). Analyzing other cytokines (Figures 4C–F and Supplementary Figure 2) significant differences were obtained in C1 compared with C2 in IL-6, CCL2 (MCP-1), and CCL3 (MIP-1alpha). Additionally, a tendential increase was observed in C1 in the chemokine IL-8.

Regarding clinical laboratory parameters, an increased tendency of inflammatory markers was observed in C1. Including markers such as C reactive protein, procalcitonin, and ferritin (Supplementary Figure 1). Moreover, relative changes in leukocyte counts were observed in C1, showing increased relative levels in neutrophils, and decreased levels in lymphocytes and platelets compared with C2 and C3 (Supplementary Figure 3).

## Discussion

SARS-CoV-2 infection is characterized by a disruption of normal immune responses, leading to uncontrolled inflammatory reactions in severe and critical patients with COVID-19 (3, 9). Immune alterations such as lymphopenia, lymphocyte activation, and dysfunction, granulocyte and monocyte abnormalities, high cytokine levels, and an increase in IgG and total antibodies are frequently observed in severe cases of COVID-19 (16) leading to dysregulated inflammation and organ failure (5). Increased systemic cytokine levels have been associated with disease severity and mortality. Different cytokines have been proposed to be key components triggering severe inflammation and multiple-organ acute injury, representing a potential dynamic tool in the prediction of the outcome of hospitalized COVID-19 patients (14, 23–26). Nevertheless, taking into consideration the complex interactions that take place in the immune system, analyzing the changes in the levels of a single cytokine might not be sufficient to understand and predict the different outcomes of this disease. Several strategies, including the analysis of multiple cytokines, have been proposed to be more useful in the prediction of severe and chronic cases of COVID-19 (19). Therefore, in the present study we used a machine learning approach to generate clusters of patients according to their cytokine profile and identified significant differences in the mortality rate among those groups.

Cluster analyses are unsupervised machine-learning algorithms that aim to delineate subgroups or clusters using a data-driven identification without the need for *a priori* labeling of datasets (27). In the context of this study, patients were divided into groups that share similar cytokine profiles, contrasting with other groups with more dissimilar patterns. Two main players were decisive in the clustering of patients, the proinflammatory chemokine CXCL10 (IP-10) and the anti-inflammatory cytokine IL-38. Significant differences in the CXCL10/IL-38 ratio was found among the groups, suggesting an important role of these two mediators in the immune responses regulating the outcome of the disease (Figure 3E). CXCL10 is a pro-inflammatory chemokine that attracts CXCR3-positive cells, such as macrophages, dendritic cells, NK cells, and activated T lymphocytes (28). High levels of CXCL10 have been reported in severe cases of COVID-19 and associated with an increased detrimental inflammatory response (29, 30). Accordingly, we report significantly elevated CXCL10 levels in the cluster with a higher mortality rate and lower survival probability (C1) (Figure 4B), suggesting that an unregulated recruitment of inflammatory cells induced by CXCL10 creates an exacerbated response that might be detrimental, leading to an increased mortality. Moreover, C1 also shows a significant increase in other inflammatory cytokines such as IL-6, CCL2, and CCL3, which have also been proposed as biomarkers associated with disease severity of COVID-19 (25, 31). However, even though CXCL10 concentrations are higher in the group with increased mortality, the concentration of this cytokine and the mortality rate showed opposite levels the clusters 2 and 3 (Figures 3C, 4B). These findings suggest that even when an exacerbated CXCL10 mediated inflammation is associated with the severity of the disease (29, 30), some degree of immune cell recruitment induced by this chemokine is necessary to overcome SARS-CoV-2 infection, accordingly to the protective role of CXCL10 reported in other coronavirus infections (32, 33).

On the other hand, increased concentrations of the anti-inflammatory cytokine IL-38 are associated with lower mortality rate among the clusters, suggesting an important role of this protein in the regulation of an adequate immune response. IL-38 is an IL-1 family cytokine (34) that regulates macrophage and T cell activation (20, 35). It has been proposed that IL-38 anti-inflammatory role, results from the interaction with two different receptors, IL-36R and IL-1RAPL1 limiting IL-17 production (20, 35–37). Moreover, IL-17 promotes neutrophil recruitment and activation, and targets pro-inflammatory genes such as CXCL10, IL-8, and CCL2 (38, 39), which are significantly (CXCL10. IL-6, CCL2, and CCL3) or tendentially (IL-8) increased in C1. Moreover, it has been shown that IL-38 reduces IL-6 secretion from macrophages (20, 37), which in combination with CXCL10 have been proposed as the central axis responsible for unleashing the cytokine storm associated with severity of the disease (40). Additionally, C1 also show an increased tendency in inflammatory markers such as CRP, Procalcitonin, and Ferritin, as well as a higher relative count of neutrophils, suggesting that the immune profile associated with the high mortality group C1 is characterized by limited IL-38-associated regulation leading to increased CXCL10 and IL-6 mediated inflammation. Interestingly, as mentioned above, cluster C3 which is characterized by the lowest mortality rate shows higher CXCL10 levels than C2, suggesting that a controlled inflammation is necessary to generate a protective immune response. C3 presents the highest levels of IL-38, indicating that possibly this anti-inflammatory cytokine is responsible for modulating the inflammatory response avoiding the harmful effects of CXCL10 mediated inflammation observed in C1. Interestingly, the CXCL10/IL38 ratio of the sink group is very similar to cluster 3 (low mortality group) but with extremely high values of IL-6. This could suggest, that in patients with an adequate CXCL10/IL-38 balance, an exacerbation of IL-6 production could increase the mortality. Unfortunately, the number of individuals in the sink group is too low to confirm this assumption. Even through, we did not detect measurable levels of IL-17 in the analyzed samples, an important limitation of our study is that we did not measure Th2 and other Th17 related cytokines.

IL-38 has been previously analyzed in serum of COVID-19 patients with some discrepancies. A study observed a negative correlation of IL-38 concentration with the severity of the disease (41), whereas a different study did not find any correlation of IL-38 with patient status (42). These differences might be associated with distinct parameters used to define disease severity, sample size and/or time point of plasma collection. In our case, even though clear differences in mortality are observed, the three clusters show a similar distribution of the clinical classification groups defined in this study, and no evident differences were observed in the frequency of symptoms and co-morbidities between the clusters, suggesting that more precise clinical parameters are necessary to define an appropriate severity classification associated with the mortality differences. Additionally, an inherent limitation associated with the type of study, is that cytokine concentrations were not analyzed in a separate group of healthy individuals to compare the concentrations reported in the clusters.

Our findings support the concept that analyzing the concentration of one single cytokine is not enough to predict the outcome of hospitalized COVID-19 patients. As it was shown for CXCL10, the changes in the concentration of this cytokine do not directly correlate with the mortality rates obtained in this study. Therefore, we outline the importance of the CXCL10/IL-38 ratio in defining the clusters of patients with different mortality rates. In summary, the balance of an inflammatory and anti-inflammatory cytokine creates three different scenarios with distinct outcomes, (i) elevated inflammatory response (high CXCL10) with deficient regulatory mechanisms (low IL-38) leads to high mortality rate (C1), (ii) reduced inflammatory response (low CXCL10) with activated regulatory mechanisms (middle IL-38) leads to middle mortality rates (C2), and (iii) moderate inflammatory response (middle CXCL10) with strong regulatory mechanisms (high IL-38) leads to low mortality rate (C3).

## Data availability statement

The original contributions presented in this study are included in the article/Supplementary material, further inquiries can be directed to the corresponding author.

## Ethics statement

The studies involving human participants were reviewed and approved by Comité Ético Científico Central CCSS Protocol R020-SABI-00262. Written informed consent for participation was not required for this study in accordance with the national legislation and the institutional requirements.

## Author contributions

AC-C, LF-P, and JM: cytokine analysis. JM-M and JM: data analysis. MS-S, CB-C, LC-F, MS-V, and JM-S: recruitment and sample collection. MR-S, DV-M, JM, AS-R, and SR-C: retrospective data collection and organization. AS-C, MS-C, JS-J, JC-F, AE-M, DL-R, JM, and AW: study design and interpretation. JM, AS-C, AC-C, and AE-M: manuscript preparation. All authors contributed to the article and approved the submitted version.

## Footnotes

1
https://www.ministeriodesalud.go.cr

2
https://www.rstudio.com/

3
https://www.r-project.org/

4
https://cran.r-project.org/web/packages/

## Supplementary material

Supplementary Figure 1 Laboratory parameters among clusters. Analysis of the indicated laboratory parameters among the clusters was performed. PT, prothrombin time; TTP, partial thromboplastin time; CRP, C-reactive protein. Statistical analysis using ANOVA and Tukey test was performed excluding the sink group. No significant differences were found.

Supplementary Figure 2 Cytokine concentrations among clusters. Analysis of the concentration values of IL-1beta **(A)**, IL-10 **(B)**, IFN-alpha **(C)**, TNF **(D)**, IL-17 **(E)**, G-CSF **(F)**, and IFN-gamma among the clusters was performed. Statistical analysis using ANOVA and Tukey test was performed excluding the sink group. No significant differences were found.

Supplementary Figure 3 Relative expression of cytokine values and laboratory parameters among the clusters. PT, prothrombin time; TTP, partial thromboplastin time; CRP, C-reactive protein.

Supplementary Figure 4 Frequencies of symptoms, risk factors and clinical outcome of the participants.

Supplementary Table 1 Demographic data and general information. BMI, body mass index; NI, not indicated; ICU, intensive care unit; ARDS, acute respiratory distress syndrome.

Supplementary Table 2 Laboratory and cytokine data. PT, prothrombin time; TTP, partial thromboplastin time; CRP, C-reactive protein.

Data sheet 1 Clustering strategies and robustness.
