# Supplementary figures and images for: Difference in mortality rates in hospitalized COVID-19 patients identified by cytokine profile clustering using a machine learning approach: An outcome prediction alternative
Source: Front Med (Lausanne). 2022 Sep 20;9:987182. doi: 10.3389/fmed.2022.987182 (PMC9530472; doi:10.3389/fmed.2022.987182)

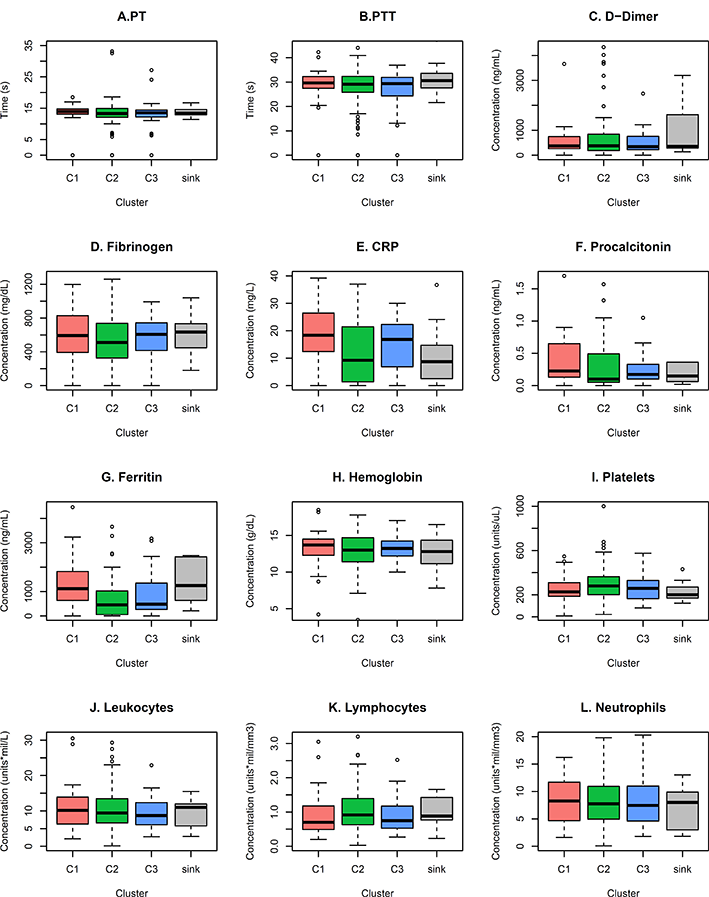

Supplement: Supplementary Figure 1 — Laboratory parameters among clusters. Analysis of the indicated laboratory parameters among the clusters was performed. PT, prothrombin time; TTP, partial thromboplastin time; CRP, C-reactive protein. Statistical analysis using ANOVA and Tukey test was performed excluding the sink group. No significant differences were found. [file Image_1.TIF]

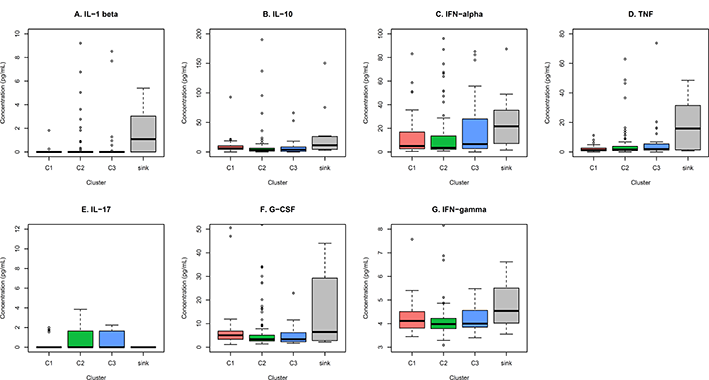

Supplement: Supplementary Figure 2 — Cytokine concentrations among clusters. Analysis of the concentration values of IL-1beta (A), IL-10 (B), IFN-alpha (C), TNF (D), IL-17 (E), G-CSF (F), and IFN-gamma among the clusters was performed. Statistical analysis using ANOVA and Tukey test was performed excluding the sink group. No significant differences were found. [file Image_2.TIF]

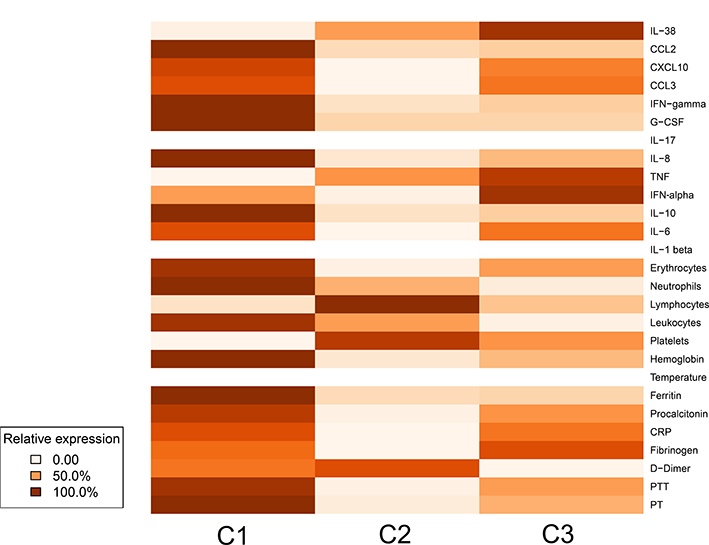

Supplement: Supplementary Figure 3 — Relative expression of cytokine values and laboratory parameters among the clusters. PT, prothrombin time; TTP, partial thromboplastin time; CRP, C-reactive protein. [file Image_3.TIF]
