# Supplementary material for: Difference in mortality rates in hospitalized COVID-19 patients identified by cytokine profile clustering using a machine learning approach: An outcome prediction alternative
Source: Front Med (Lausanne). 2022 Sep 20;9:987182. doi: 10.3389/fmed.2022.987182 (PMC9530472; doi:10.3389/fmed.2022.987182)
Supplement: Supplementary Figure 4 — Frequencies of symptoms, risk factors and clinical outcome of the participants. [file Image_4.pdf]

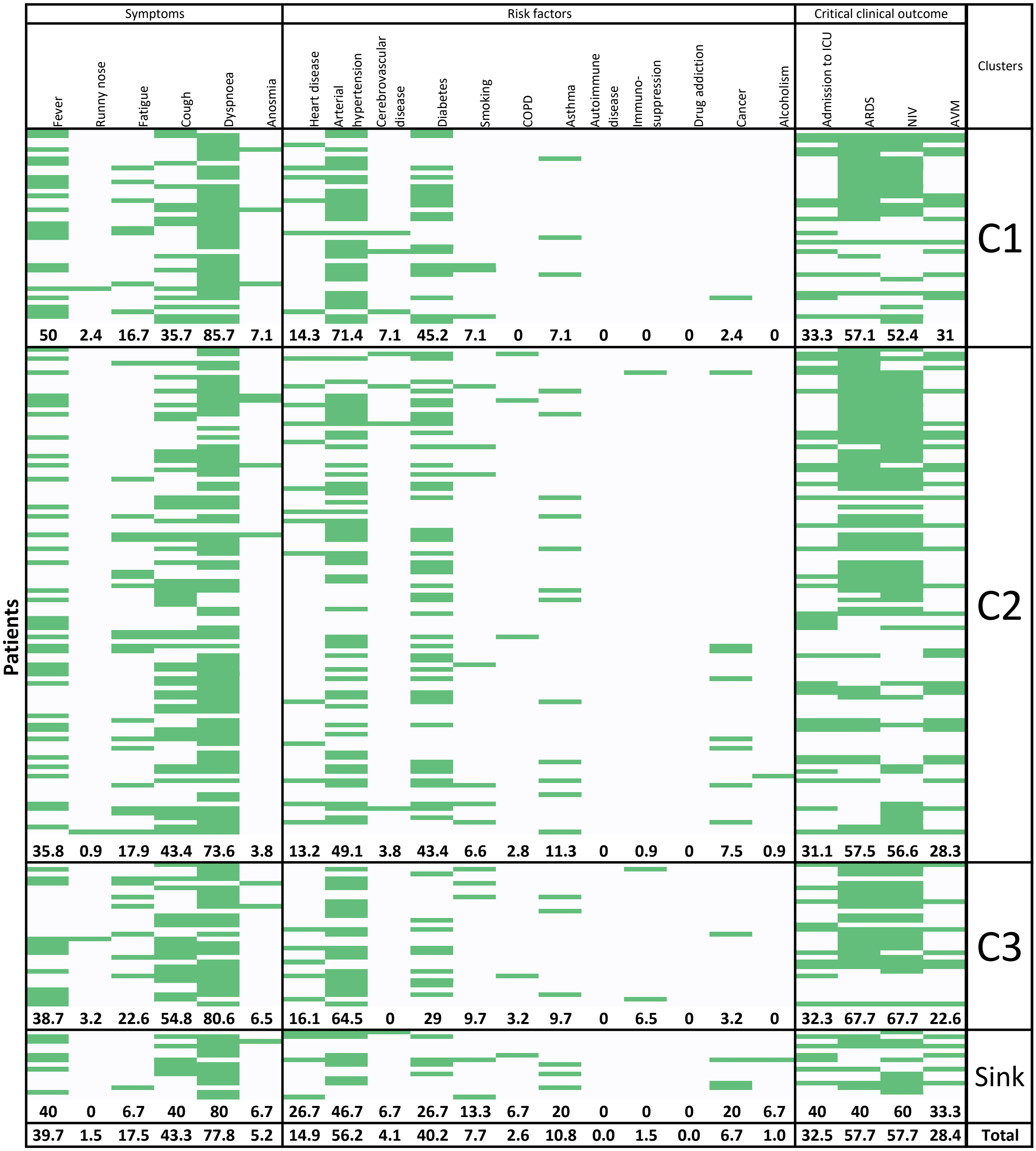
Symptoms
Risk factors
Critical clinical outcome
Fever
Runny nose
Fatigue
Cough
Dyspnoea
Anosmia
Heart disease
Arterial hypertension
Cerebrovascular disease
Diabetes
Smoking
COPD
Asthma
Autoimmune disease
Immuno-suppression
Drug addiction
Cancer
Alcoholism
Admission to ICU
ARDS
NIV
AVM
Clusters
Patients
C1
50 2.4 16.7 35.7 85.7 7.1 14.3 71.4 7.1 45.2 7.1 0 7.1 0 0 0 2.4 0 33.3 57.1 52.4 31
C2
35.8 0.9 17.9 43.4 73.6 3.8 13.2 49.1 3.8 43.4 6.6 2.8 11.3 0 0.9 0 7.5 0.9 31.1 57.5 56.6 28.3
C3
38.7 3.2 22.6 54.8 80.6 6.5 16.1 64.5 0 29 9.7 3.2 9.7 0 6.5 0 3.2 0 32.3 67.7 67.7 22.6
Sink
40 0 6.7 40 80 6.7 26.7 46.7 6.7 26.7 13.3 6.7 20 0 0 0 20 6.7 40 40 60 33.3
Total
39.7 1.5 17.5 43.3 77.8 5.2 14.9 56.2 4.1 40.2 7.7 2.6 10.8 0.0 1.5 0.0 6.7 1.0 32.5 57.7 57.7 28.4
