# Supplementary material for: Difference in mortality rates in hospitalized COVID-19 patients identified by cytokine profile clustering using a machine learning approach: An outcome prediction alternative
Source: Front Med (Lausanne). 2022 Sep 20;9:987182. doi: 10.3389/fmed.2022.987182 (PMC9530472; doi:10.3389/fmed.2022.987182)
Supplement: Data sheet 1 — Clustering strategies and robustness. [file Data_Sheet_1.PDF]

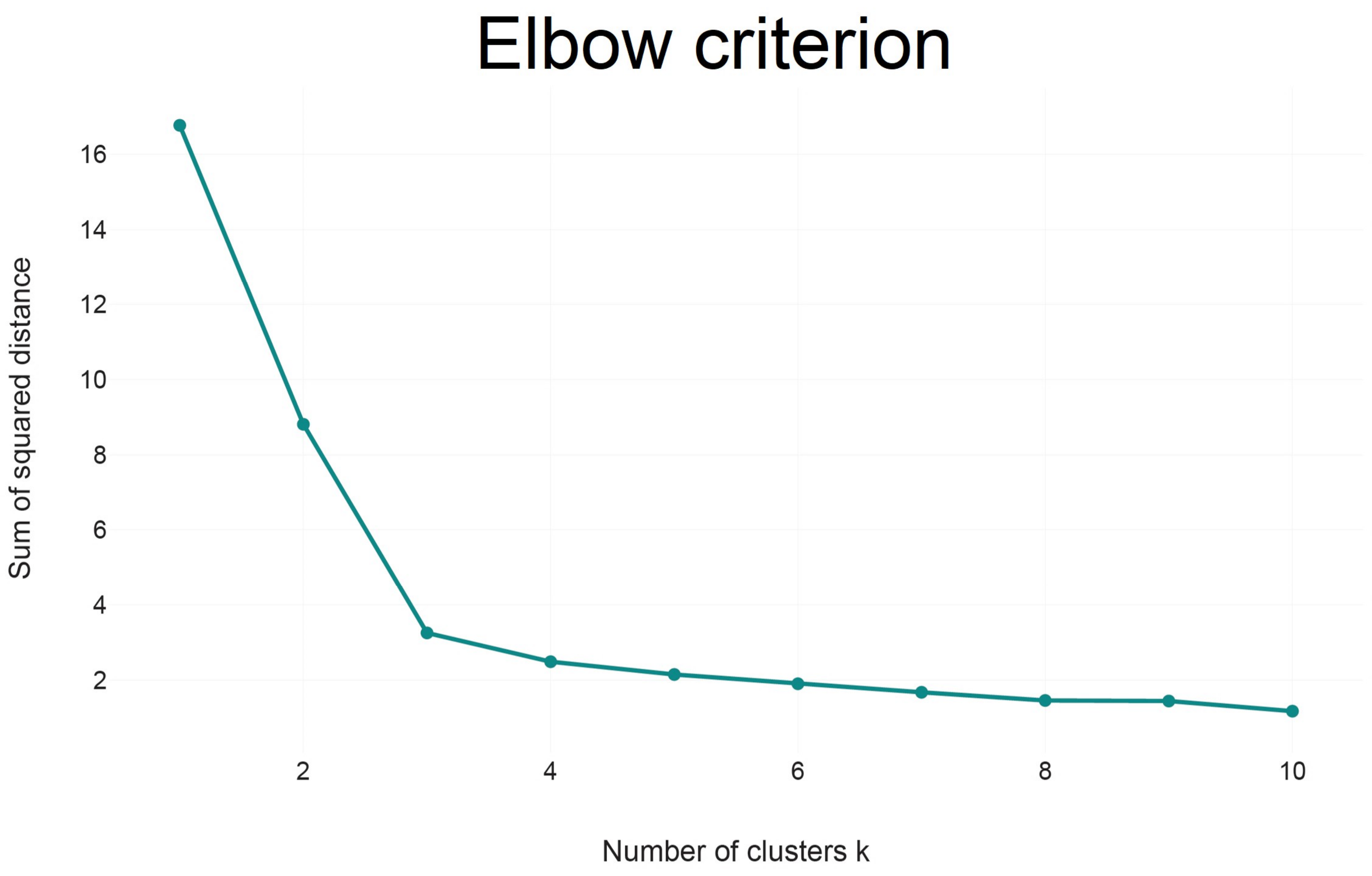
Elbow criterion
Sum of squared distance
16
14
12
10
8
6
4
2
2
4
6
8
10
Number of clusters k

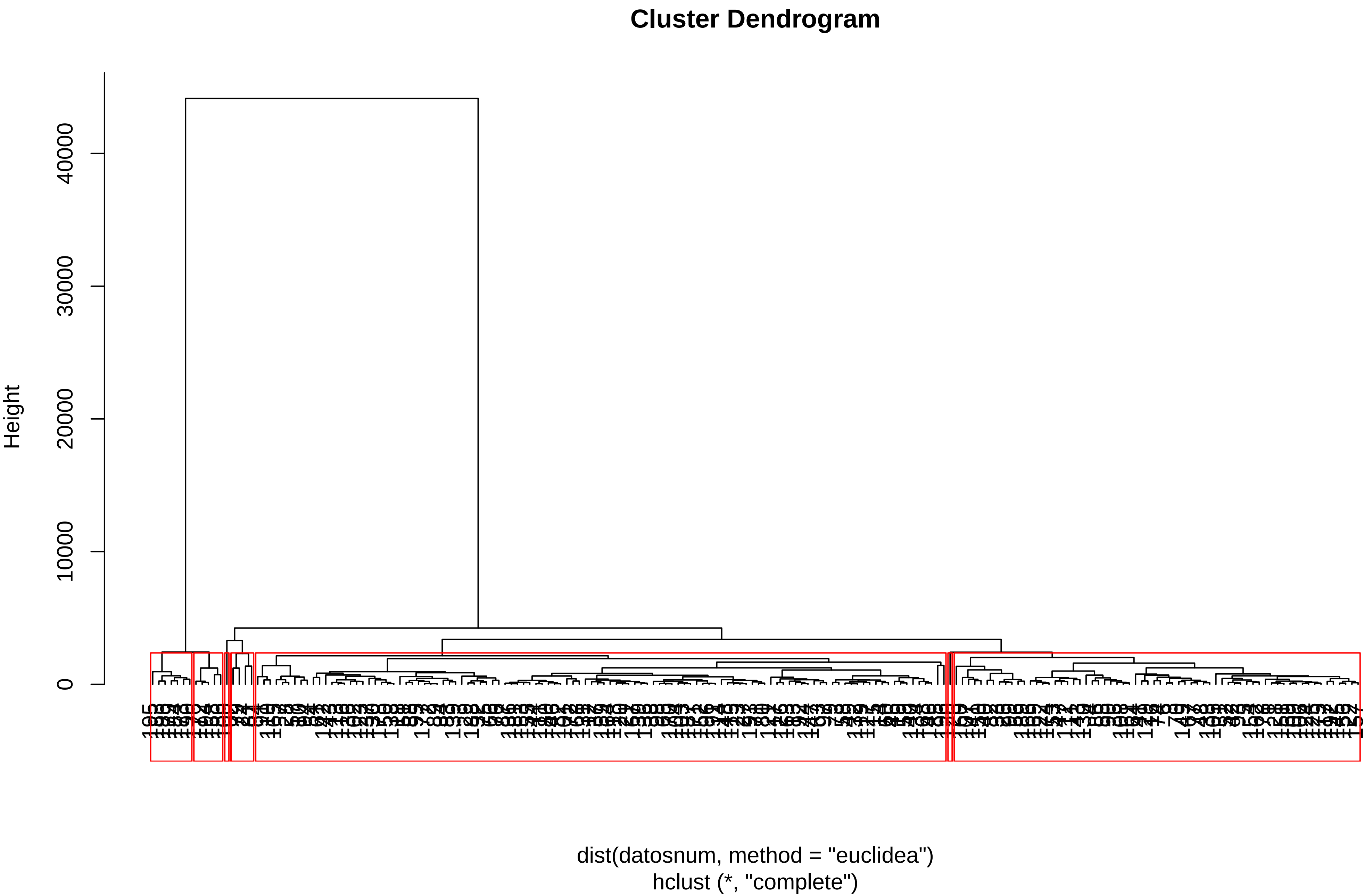

Cluster Dendrogram
Height
0
10000
20000
30000
40000
dist(datosnum, method = "euclidea")
hclust (*, "complete")

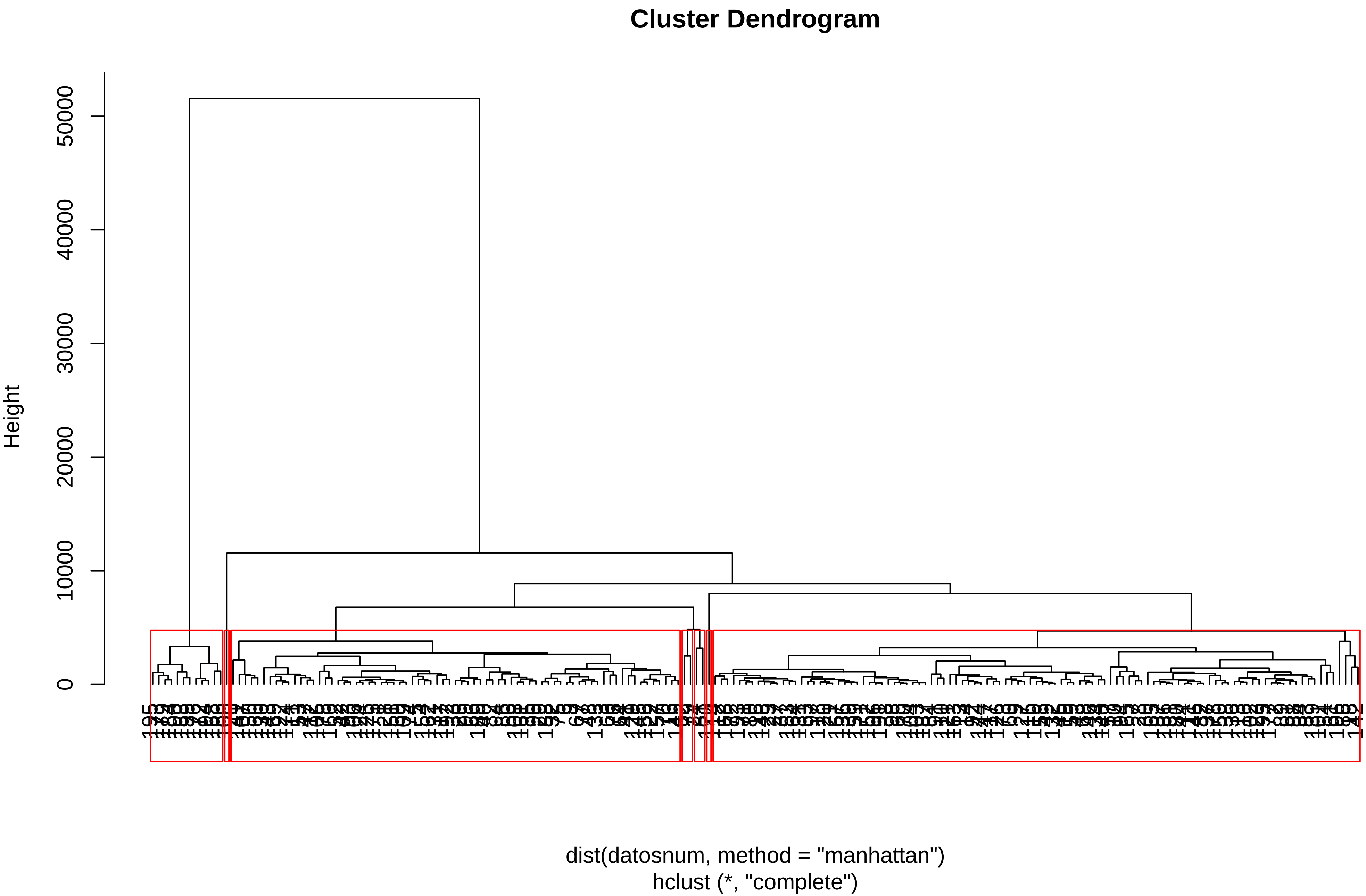
Cluster Dendrogram
Height
0
10000
20000
30000
40000
50000
dist(datosnum, method = "manhattan")
hclust (*, "complete")

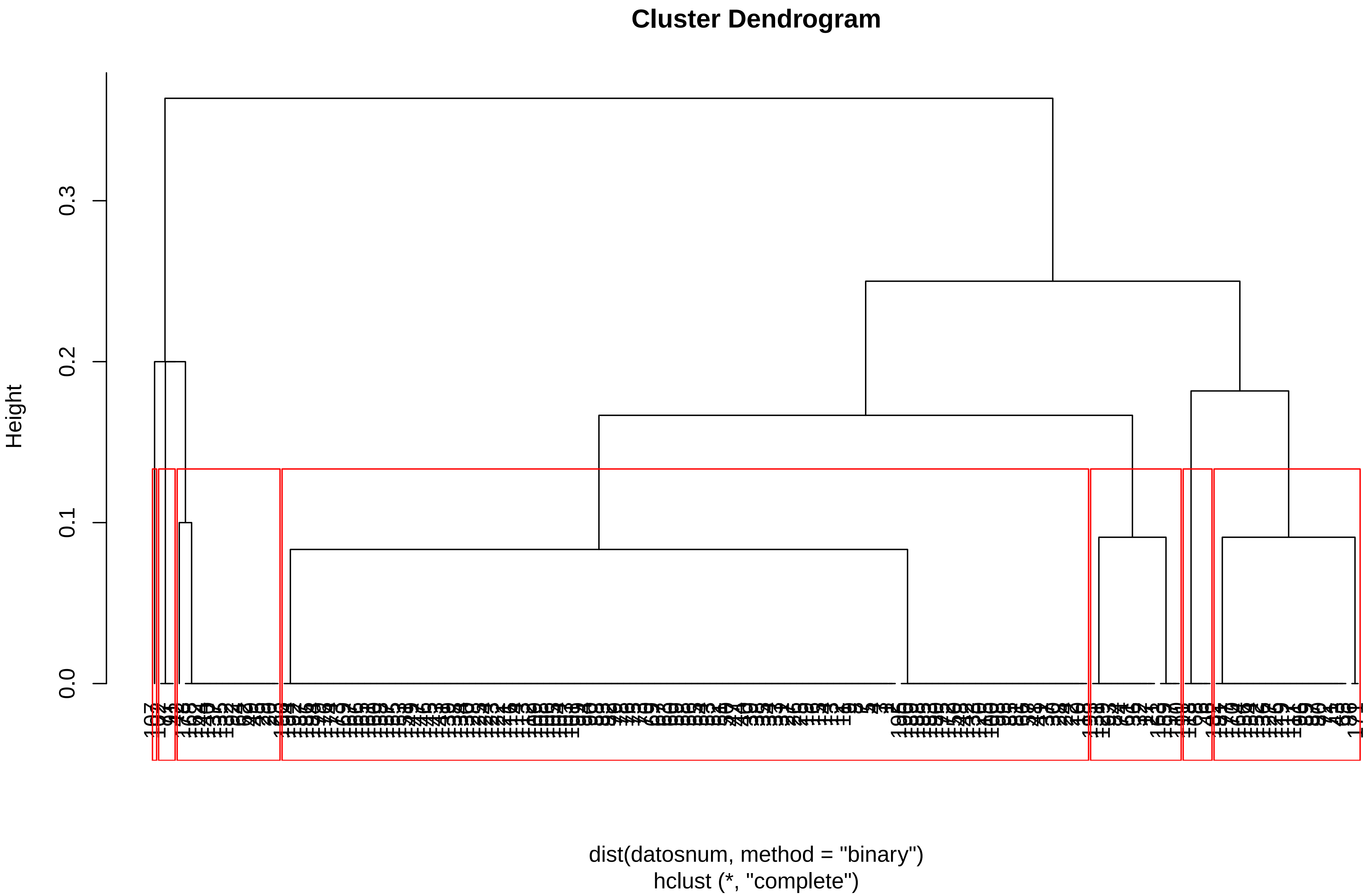
Cluster Dendrogram
Height
0.0
0.1
0.2
0.3
dist(datosnum, method = "binary")
hclust (*, "complete")

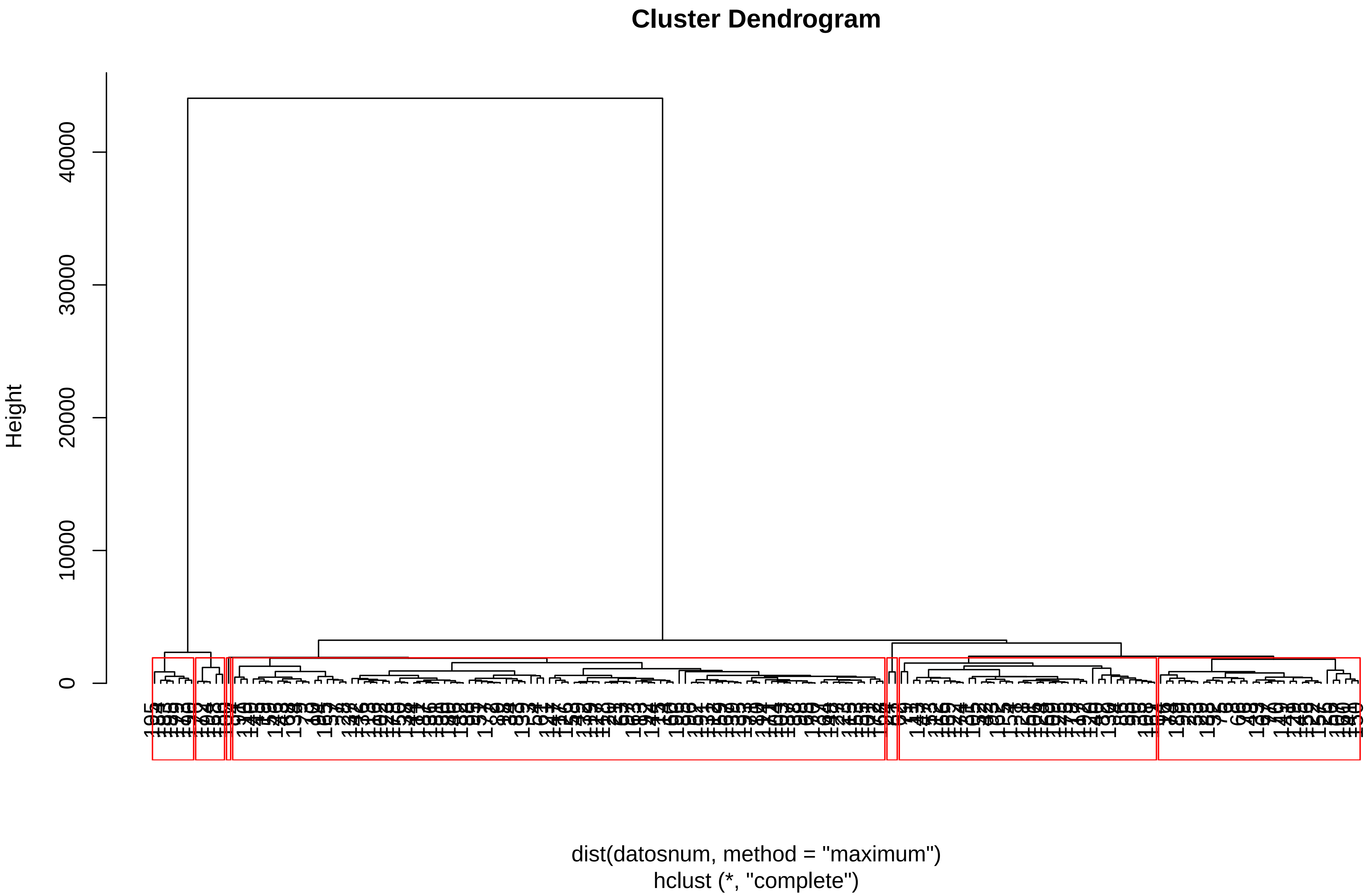
Cluster Dendrogram
Height
0
10000
20000
30000
40000
dist(datosnum, method = "maximum")
hclust (*, "complete")

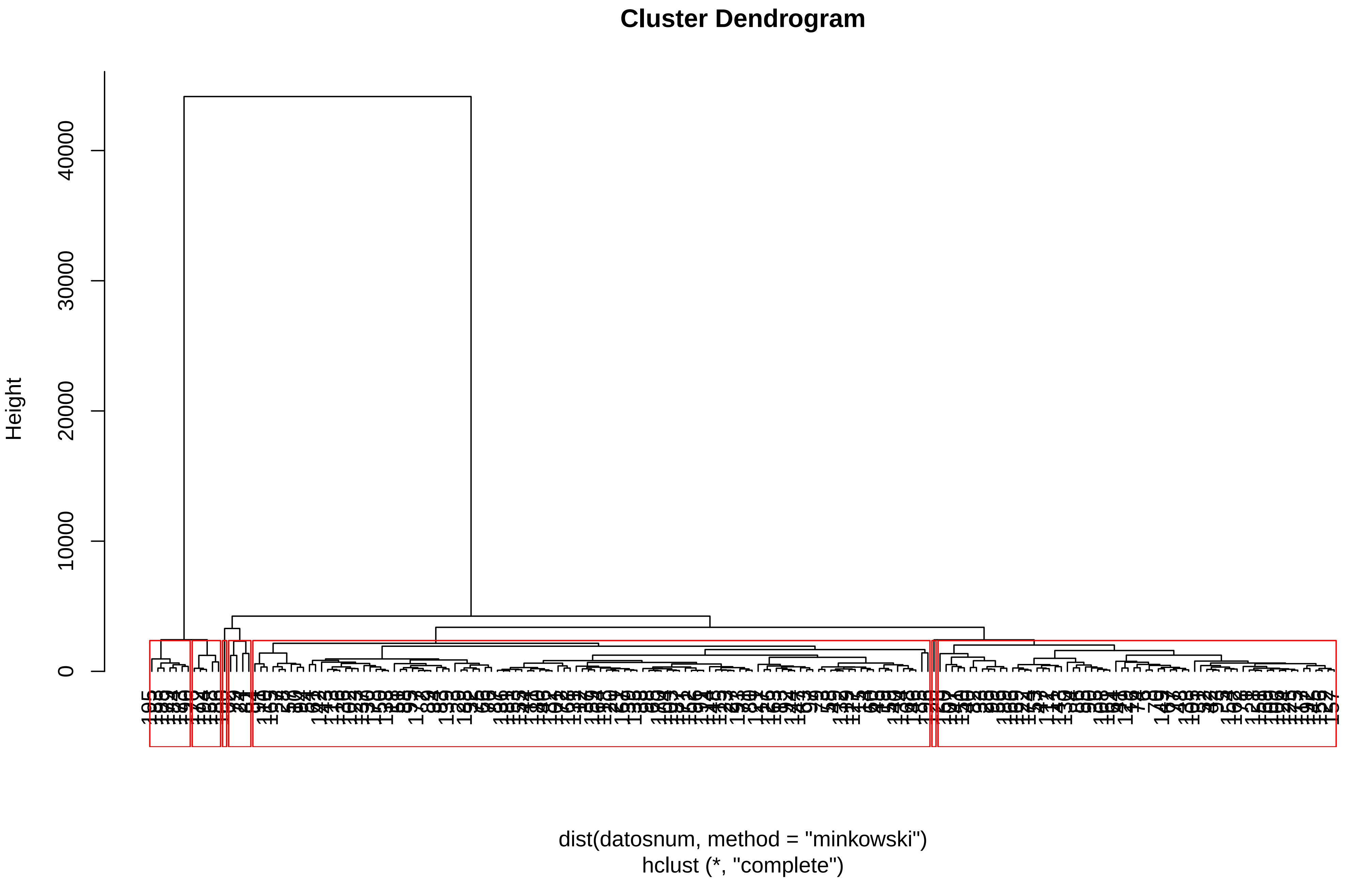
Cluster Dendrogram
Height
0
10000
20000
30000
40000
dist(datosnum, method = "minkowski")
hclust (*, "complete")
